# Assessment of the Impact of Accelerated Migration Testing for Coated Food Cans Using Food Simulants

**DOI:** 10.3390/molecules24173123

**Published:** 2019-08-28

**Authors:** Rafael Paseiro-Cerrato, Lowri DeJager, Timothy H. Begley

**Affiliations:** US FDA, Center for Food Safety and Applied Nutrition, 5001 Campus Drive, College Park, MD 20740, USA

**Keywords:** migration, coatings, polyester, acrylic-phenolic, epoxy, vinyl, food cans, monomers, oligomers, non-intentionally added substances (NIAS)

## Abstract

In this study, an accelerated migration test on food can coatings into food simulants was investigated. Food simulants covering a wide range of polarity were used to conduct migration tests at 60 °C with storage times ranging from 4 h to 30 days. Epoxy-resins, acrylic–phenolic, polyester, and vinyl coatings were exposed to water, 3% acetic acid, 50% ethanol, and Miglyol 812^®^. Using liquid chromatography coupled to a variety of detectors (UHPLC-Q-Orbitrap-MS, UFLC-MS/MS, and HPLC-DAD), migration of several monomers and previously identified oligomers, as well as some unidentified migrants, were determined during the experiment. The data from this study was compared to our findings from previous long-term migration studies with storage times ranging from 24 h to 540 days at 40 °C using the same can coating applications. The results illustrate that performing migration experiments for short time periods at 60 °C may mimic migration results that would be obtained at 40 °C after long-term migration tests (up to 1.5 years) from food can coatings into food simulants.

## 1. Introduction

It is well-known that food contact materials (FCM) may transfer some of their constituents into food. This mass transfer may increase when the products are exposed to high temperatures (e.g., retorted or microwavable products) and longer time periods. Government agencies usually regulate the components of FCM in food packaging products. In order to determine the concentration of components that can migrate into food, it is sometimes required to analyze the target compounds in foods. Currently, the most commonly used analytical techniques for identifying and analyzing small migrating molecules from FCM are based in liquid or gas chromatography [1,2,3,4,5,6,7,8,9,10]. However, the determination of migrants in actual food can be a real challenge, because of the different food varieties (fatty food, aqueous food, acidic food, etc.), matrix interferences in the analysis, and challenges in the extraction. Furthermore, food analyses are time consuming and costly. To facilitate the determination of migration from FCM, food simulants are commonly used, which are usually mixtures of water, organic solvents, and acids, as well as oils (e.g., 3% acetic acid, mixtures ethanol/water, vegetable oils). The analysis of food simulants is generally more straightforward than real food. Ideally these simulants mimic real food and therefore, estimate migration. Some of the first migration studies from FCM into food simulants were performed during the 1970s and 1980s [11,12,13,14,15,16]. In the subsequent years, many publications have determined the migration from FCM into food simulants. However, most of the tested FCM used for these investigations were plastic materials [17,18,19,20,21,22,23,24,25,26,27,28,29] rather than other FCM such as coatings.

When performing migration studies, the test temperature is usually above the food package end use temperature. In addition, for migration tests involving prolonged storage conditions, the test times and temperature applied during the test should mimic the expected storage conditions for the end product. For plastics, the most common migration test is 10 days at 40 °C, however, this may not be adequate for some FCM that are exposed to high temperature during use or are thermally treated. For example, microwavable packaging may reach temperatures higher than 200 °C [17,30]. The period of time that the FCM is in contact with food may also vary depending on the FCM and/or the type of food. For example, foods that have low water content (e.g., flour), also have lower water activity resulting in a long shelf life due to decreased biological activity of microorganisms in these foods [31]. 

Food can coatings may be in contact with food for years (2–5 years). Recently, several publications on different can coatings and food simulants indicate that concentrations of migrants continue to increase after traditional migration testing in different types of can coatings using food simulants. Vaclavikova et al. [32], performed migration studies using polyvinyl chloride (PVC) can coatings into 3% acetic acid and water and found benzoguanamine (BGA) concentrations continued to increase even after 1 year. Increases in monomer and oligomer concentrations were also observed from polyester, epoxy, and acrylic–phenolic coatings for food contact applications into food simulants [33,34]. Changes beyond the traditional migration test (10 days at 40 °C) were observed in all coatings under at least one testing condition. From these experiments it was concluded that the traditional test conditions for extended storage may not accurately predict migration for extended time periods in can coatings. 

The European Commission regulation 10/2011 for plastic materials [35], specifies that plastic FCM stored with food for periods longer than 6 months should use migration testing at 60 °C for 10 days. Even though this regulation is just for plastic materials, it is often used as reference or guidelines for other non-plastic FCM (e.g., adhesives, coatings).

This investigation was designed to evaluate the applicability of an accelerated migration test to predict long-term migration from commonly used food can coatings into food simulants. Four cases (polyester, acrylic–phenolic, vinyl, and epoxy—resins) are taken and discussed as typical or representative can coating applications. The selected testing temperature was 60 °C with test time periods of up to 30 days. The presented data may support considerations on the appropriate testing of can coatings.

## 2. Results and Discussion 

### 2.1. Epoxy Coatings

**Figure 1:**
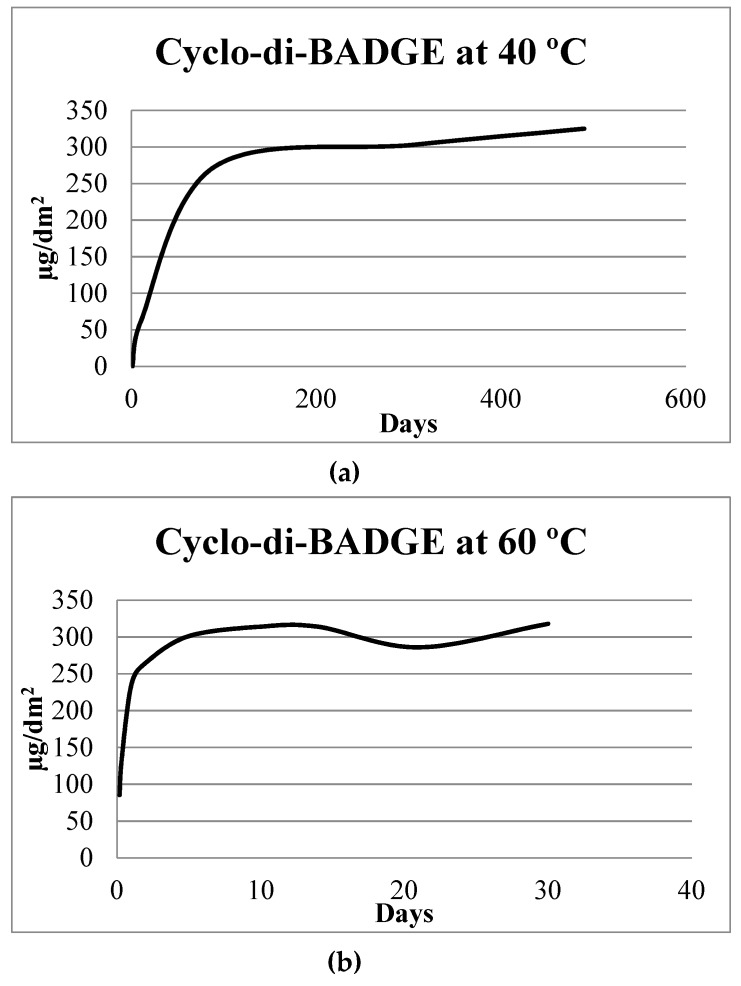
Comparison of migration into 50% ethanol of cyclo-di BADGE at 40 °C (**a**) (data obtained from [34]) and at the accelerated test performed in this study at 60 °C (**b**).

Migration test data into 50% ethanol from epoxy cans (60 °C for 30 days) are presented in Table 1. These data illustrate that mass transfer was completed at or before 10 days for monomers and most of epoxy oligomer derivatives. Afterward, in most cases the compounds remained chemically stable during the migration test up to 30 days in the simulant. Previous studies have demonstrated that migration tests of 10 days at 40 °C could not estimate migration for a long-term exposure for can storage [34]. Generally, the concentrations observed in the current migration studies at 10 days and 60 °C, are equivalent to the long-term migration experiments at 40 °C obtained by Paseiro-Cerrato et al. [34]. These data are illustrated for cyclo-di-BADGE at 40 °C and 60 °C in Figure 1. For the cyclo-di-BADGE migration, concentrations at 10 days (314 µg/dm^2^) were comparable to the levels obtained for 490 days at 40 °C (325 µg/dm^2^), which means that the acceleration factor was 49. On the other hand, when monitoring the unknowns 1 and 2 (most likely non-epoxy related derivatives described by Paseiro-Cerrato et al. [34]), we observed that migration slightly increased beyond ten days at 60 °C (see Table 1). The concentrations levels equivalent to those reported by Paseiro-Cerrato et al. [34] were reached after 21 days at 60 °C. These migration concentrations were compared using the same analytical and instrumental methods. It is important to state that at 10 days the simulant appeared to acquire a slight yellow color. No visual change in the coating structure was observed after the migration test, which was expected. As can coatings are typically cured at very high temperatures and are retorted at temperatures above 100 °C, this visual change maybe an indication of migration of UV-vis active substances from the coating. 

For the epoxy coatings in contact with Miglyol 812^®^, a subset of the cans was subjected to a retort step. To simulate a real retort process, heating conditions should result in internal temperatures of 127 °C. Appropriate heating times were determined using cans filled almost to the top, in order to allow expansion of the liquid, with Miglyol 812^®^. Cans were heated, and the oil temperature was measured at different time intervals. It was determined that oil reached 124 °C in 75 min, therefore, 105 min was considered an appropriate time to mimic retort conditions employed by industry [34]. Migration levels obtained into Miglyol 812^®^ from the migration test are listed in Table 2. The data illustrate that after the retort step mass transfer appears to be complete. Analyte concentrations remained constant during the rest of the experiment. 

Accelerated migration tests of the epoxy coatings in contact with Miglyol 812^®^ that were not subjected to a retort step (non-retorted cans) did not have detected oligomers in the Miglyol 812^®^. Similar results were obtained using fatty food simulant such as isooctane in the study by Paseiro-Cerrato et al. [34]. Only very low concentrations of bisphenol A (BPA) were measured in the Miglyol 812^®^. In this case, the partitioning equilibrium was reached at 10 days migration at 60 °C, where a concentration of 0.7 ± 0.01 µg/dm^2^ of BPA was obtained.

### 2.2. Acrylic–Phenolic Coating

**Figure 2:**
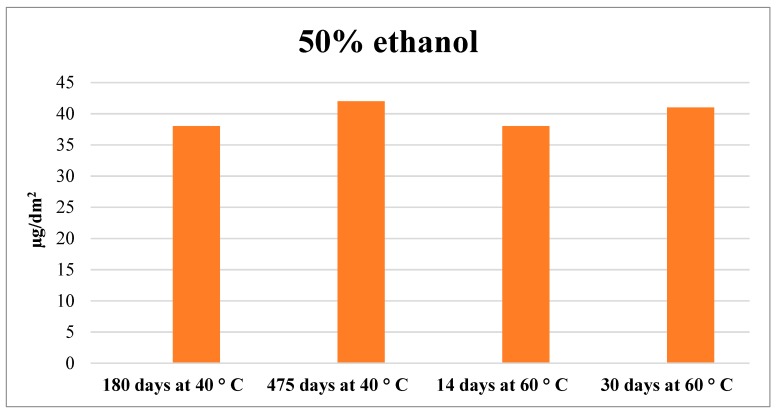
Comparison of migration into 50% ethanol of unknown E at 40 °C (data obtained from Paseiro-Cerrato et al. [34]) and at the accelerated test performed in this study at 60 °C.

Migration studies at 60 °C of acrylic–phenolic coatings into 50% ethanol found that the migration of benzoguanamine (BGA) and unknown B (migrant described by Paseiro-Cerrato et al. [34]) into food simulant gradually increased up to 30 days. These results are listed in Table 3. Continued migration for up to 30 days could be due to a degradation process of some higher molecular weight migrants with time. This statement is supported by previously published data using the same can coating application [34], where concentrations in the acetonitrile total coating extract were lower than migration concentrations in the simulants. Concentrations of unknown E, once the mass transfer was completed, were consistent with results obtained at 40 °C for 1.5 years. This is illustrated Figure 2. The concentration of unknown E in the simulant at 40 °C after the long-term migration experiment was 42 µg/dm^2^ and at 60 °C at 30 days 41 µg/dm^2^ was measured. Similar results were observed for BGA and the rest of tracked compounds. The data presented here suggest that appropriate short time periods at 60 °C would mimic long-term migration times. The acceleration factor in the accelerated migration test would be 15.

### 2.3. Polyester Coating

**Figure 3:**
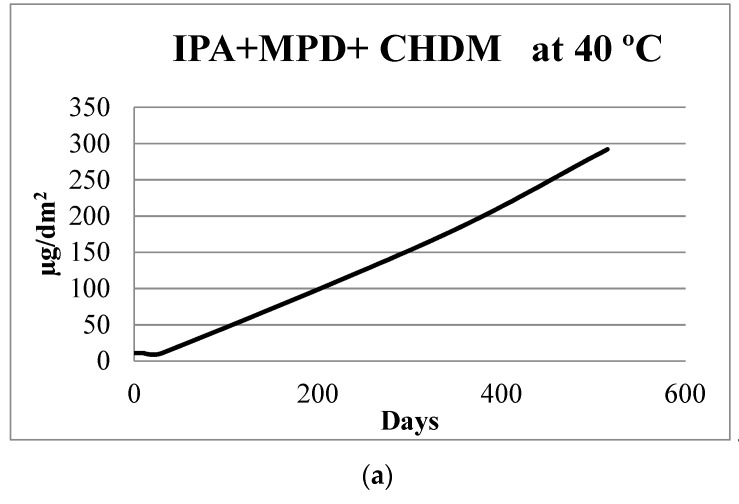

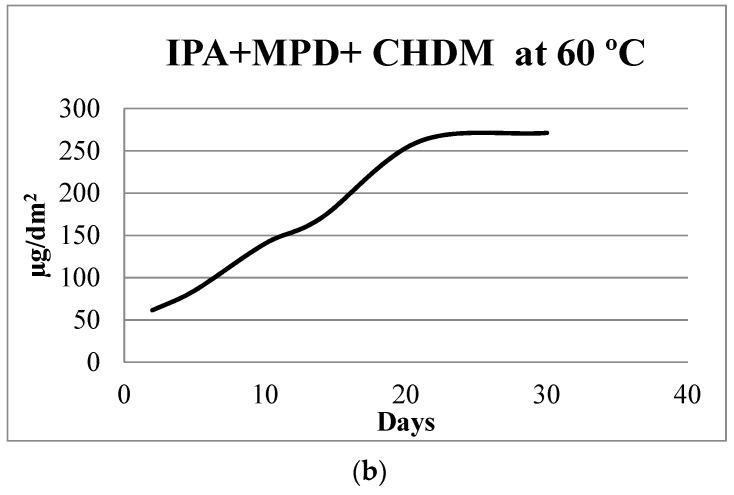
Comparison of migration into 50% ethanol of a polyester oligomer IPA + MPD + CHDM at 40 °C (**a**) (data obtained from Paseiro-Cerrato et al. [33]) and at the accelerated test performed in this study at 60 °C (**b**). IPA + MPD + CHDM is listed as IPA + MBO + COH in Paseiro-Cerrato et al. [33].

For polyester can coatings, results of migration in 50% ethanol are listed in Table 4 and have concentrations increasing after 10 days for most of the compounds. The unknowns are unidentified migrants (most likely polyester oligomers), but they supply important information about the performance of the polyesters during the migration experiment. Oligomers migration increase up to 21 days and then statistically remain stable. When comparing data with DAD results from Paseiro-Cerrato et al. [33], the current data illustrate similar concentrations at the end of this study for most of the compounds. An example of comparable results obtained at the end of the long migration test at 40 °C is illustrated in Figure 3. This suggests that the 60 °C accelerated test achieves closer estimates of the worst-case migration than a migration scenario at 40 °C (acceleration factor of 17). It is important to note that some compounds show a small tendency to decrease in concentrations after 21 days of exposure although these decreases in concentrations are not statistically significant. For example, IPA + 2 MPD decreases from 169 at 21 days to 155 µg/dm^2^ at 30 days. As a hydrolysis process could occur for polyester derivatives, it could be possible that longer test times may underestimate exposure concentrations for some polyester components.

In Figure 3, it can be also observed that the IPA + MPD + CHDM achieved a plateau at 60 °C while in the test at 40 °C it did not. We propose three hypotheses to explain this phenomena. The hydrolysis process that may create this oligomer has stopped because higher molecular weight (MW) oligomer(s) have been completely hydrolyzed. Another explanation could be that the oligomer itself is also suffering from hydrolysis, being hydrolyzed into other smaller MW oligomers. Only a standard of the oligomer could prove the two suggested hypotheses presented above. Finally, the interaction between the simulant and the polymer, that could be also involved in the diffusion of the migrant as described previously [33], could have come to an end.

### 2.4. Vinyl Coating

**Figure 4:**
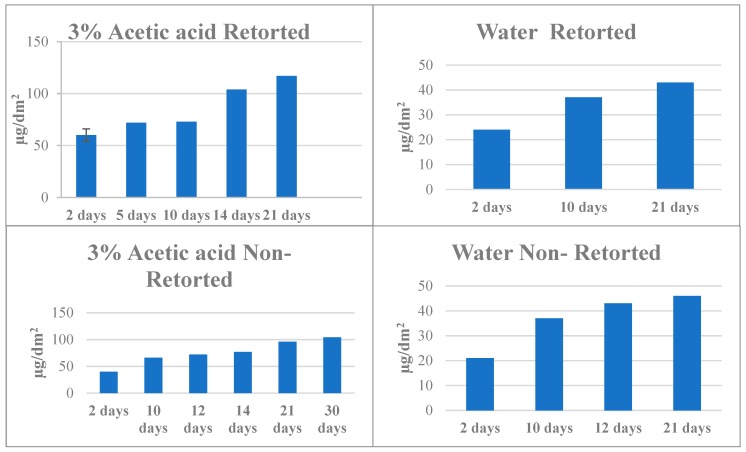
Concentrations of BGA from vinyl coatings in water and 3% acetic acid food simulant at 60 °C during the accelerated migration test. Results expressed in µg/dm^2^.

For the analyte BGA migrating from vinyl coatings into aqueous food simulants, similar mass transfer results were obtained in the 60 °C accelerated test as with the three previously studied coatings. Concentrations increased with time in all test food simulants (see Figure 4). These data were consistent with tests reported by Vaclavikova et.al. [32]. Migration from the 1.5-year long-term test was similar to that obtained at 10–14 days at 60 °C, suggesting that the accelerated test would be more appropriate for estimating migration for long-term exposure in a short period of time.

## 3. Materials and Methods

### 3.1. Chemicals

Acetonitrile (ACN) (Optima^®^ for LC-MS), water (Optima^®^ for LC-MS), and hexanes were obtained from Fisher Scientific (Fair Lawn, NJ, USA). Ethyl alcohol absolute 200 Proof, 99.5+% was purchased from Acros Organics (Fair Lawn, NJ, USA). Distilled white vinegar was supplied by an industrial partner. Miglyol 812^®^ was purchased from Cremer Oleo Division (Hamburg, Germany). Bisphenol A (ring ^13^C_12_) (BPA-C13), 99% was purchased from Cambridge Isotope Laboratories, Inc. (Andover, MA, USA). 2,4-Diamino-6-phenyl-1,3,5-triazine (benzoguanamine, (BGA)) 97%, Bisphenol A ≥ 99% (BPA) and Bisphenol A –*d*16 98 atom % D (BPA-*d*16), were purchased from Aldrich (St. Louis, MO, USA). N,N-Bis-(2-hydroxyethyl) terephthalate (BHET) was acquired from Polysciences Inc. Bisphenol A diglycidyl ether ≥ 95% (BADGE), Bisphenol A(2,3-dihydroxypropyl) glycidyl ether ≥ 95% (BADGE.H2O), Bisphenol A bis (2,3-dihydroxypropyl) ether ≥ 97% (BADGE.2 H2O), formic acid for LC-MS ≥ 98%, and ammonium formate for mass spectrometry ≥ 99.0% were purchased from Fluka (St. Louis, MO, USA). 

### 3.2. Migration Experiment

All cans were generously provided by industrial partners. The cans were lined with epoxy-resins, acrylic–phenolic, polyester, and vinyl coatings. Retorted (100 °C for 27 min) and not-retorted vinyl cans containing water and 3% acetic acid were filled and sealed at industry facilities. Vinyl cans were stored at ambient temperature (15.6–28.3 °C) and analyzed. A set of cans lined with polyester, acrylic–phenolic, and epoxy-resin coatings were filled with 50% degassed ethanol and spared with nitrogen before sealing. In addition, a set of cans containing the epoxy coating were filled with Miglyol 812^®^ and sealed. A group of cans containing Miglyol 812^®^, were placed into an oven at 127 °C for 1 h 45 min to mimic retort conditions applied by industry, the other group was not subjected to the thermal treatment (non-retorted). Cans were stored at 60 °C for different times, 0.17, 0.33, 1, 2, 5, 10, 12, 14, 21, and 30 days. For every type of coatings, a migration test(s) was performed in triplicate to obtain statistically significant data. Recovery experiments (n = 5) for BPA in Miglyol 812^®^, were performed at 15 ng/g and 240 ng/g, and the results ranged from 98% ± 3% to 102% ± 7%. Aqueous food simulants were stored until analysis in glass vials, capped with a PTFE septa, and polypropylene caps and stored in the refrigerator (1–8 °C). Miglyol 812^®^ samples were stored at laboratory temperature (20 °C) and protected from light.

### 3.3. Method Development

Fifty percent water/ethanol food simulant was directly analyzed by high performance liquid chromatograph coupled to a diode array detector (HPLC-DAD) to determine oligomers and unknowns previously tracked in migration experiments [33,34]. Ultra-fast liquid chromatograph coupled to a mass spectrometer (UFLC-MS/MS) was used for BPA analysis. Samples were spiked with an internal standard prior to analysis. For BGA determination, an ultra-high performance liquid chromatograph coupled to a high resolution mass spectrometer (UHPLC-Q-Orbitrap-MS) was used. For vinyl cans containing aqueous food simulants, 950 µL of water was added to 50 µL of the simulants before the analysis.

For Miglyol 812^®^ analysis, 7 mL of hexane and 7.5 mL acetonitrile were added to 0.5 g of Miglyol 812^®^ spiked with BPA-C13 and accurately weighed into a glass vial. The samples were capped with a PTFE septa and polypropylene cap, and agitated at 1000 rpm for 1.5 min. The acetonitrile solution was removed and concentrated to dryness under a nitrogen stream. Afterwards, 0.75 mL of acetonitrile was added, followed by 0.75 mL of water and 0.25 mL of hexane. The solution was shaken in an agitator (Glas-Col^®^) for 30 sec at 1000 rpm. After 5 min, two phases appeared to be separated and 1 mL of the acetonitrile/water solution was extracted. A second extraction with 0.5 mL of acetonitrile followed by 0.5 mL of water and 0.25 mL of hexane was performed. One mL of the extract was then spiked with internal standard and analyzed by HPLC-DAD and UFLC-MS/MS.

### 3.4. Instrumentation

A Shimadzu UFLC XR equipped with a degasser DGU-20A3, two pumps LC-20AD XR, an auto sampler SIL-20AC XR, column oven CTO-20AC, and a communications bus module CBM-20A was coupled to a Sciex 5500 Qtrap and controlled by Analyst software. For BPA, the separation method was as follows: an Acquity UPLC^®^ BEH C18 1.7 µm, 2.1 × 150 mm column coupled to a Vanguard^TM^ BEH C18 1.7 µm, and 2.1 × 5 mm column was used with a gradient starting at 50:50 acetonitrile/water for 2 min and increasing to 100% acetonitrile in 4 min. The mobile phase was maintained for another four minutes to clean the column. Flow was 0.2 mL/min, autosampler temperature 15 °C, injection volume was 5 µL, and oven temperature was 40 °C. Detector settings in the negative mode were: ion spay voltage −4500 V, temperature 450 °C, CAD was set at high, curtain gas set at 30, declustering potential was −55, ion source gas 1 was 60, and ion source gas 2 was 90 entrance potential was −10. BPA transitions were 227.3/212.3 (CE −28, CXP −13) and 227.3/133.1 (CE −36, CXP −13), BPA-C13 transitions were 239.0/224.0 (CE −27, CXP −12) and 239.0/139.0 (CE −36, CXP −14), and BPA-*d*16 was 241.1/142.2 (CE −36, CXP −12).

BGA, BADGE, BADGE.H_2_O, BADGE.2H_2_O, and previously identified oligomers as well as migrants with unknown nature tracked in a previous migration experiment [34], were determined using the method described in that experiment and another conducted by Vaclavikova et al. [32]. For analysis of 50% ethanol, the same HPLC-DAD conditions described by Paseiro-Cerrato et al. [33,34] were used.

Oligomers determinations from the Miglyol 812^®^ extracts were conducted using the same HPLC-DAD system. Mobile phases were water (A) and acetonitrile (B). Gradient started with 50% B and gradually increased to 60% in 6 min, then raised to 100% in 10 min and held for 7 min. A stepwise return of 5 min to initial concentration was used for column equilibration. Flow rate was 0.3 mL/min and injection volume was 10 µL. The stationary phase was an Agilent Zorbax Extend C18, 80 Å (2.1 × 150 mm, i.d 3 µm) thermostatted at 40 °C. Migrants were detected at 232 nm in the DAD.

All chromatographic methods were validated in terms of limit of detection (LOD), limit of quantification (LOQ), % relative standards deviation (RSD), and correlation coefficient, see Table 5. For the analysis of oligomers and unknown compounds, proxy standards including BHET, BADGE, and BADGE.2H_2_O were used for polyesters, epoxy, and acrylic–phenolic coatings, respectively, as it was suggested elsewhere [34,36,37,38,39].

## 4. Conclusions

The results obtained in these experiments show that for the studied coatings, migration tests at 60 °C could be used to simulate long-term can storage and provides similar results to long-term studies (up to 1.5 years) in food simulants at 40 °C in shorter times (monitoring multiple time points from 4 h to 30 days). The obtained acceleration factors ranged from 15 to 50 using the test at 60 °C. Mass transfer of compounds from epoxy-coatings in 50% ethanol at 60 °C is completed at ten days for most analytes. Due to their chemical stability, migration experiments using epoxy-derivatives did not demonstrate significant concentration changes. Studies of polyesters coatings show that most oligomers increase during the experiment. A similar behavior is observed for acrylic–phenolic and vinyl coatings

For some of the analyzed migrants, 60 °C for 10 days seems to mimic migration results obtained in previous studies at 40 °C up to 1.5 years, this is especially true for epoxy coatings. However, for certain compounds, particularly migrants from polyester and acrylic coatings used in this study, testing times longer than 10 days would be required. On the other hand, increasing migration times at 60 °C could result in hydrolysis and therefore underestimate the exposure to some migrating components. EU regulation 10/2011 in plastic materials [35] specifies that for plastic FCM in contact with food for extended periods of times (above 6 months), a test at 60 °C for 10 days should be performed. A migration test at 60 °C monitoring multiple time points from 4 h to 10 days could be used as a compromise to estimate migration from can coatings into food simulants after a long-term exposure.

## Figures and Tables

**Table 1:** Concentrations of tracked migrants from epoxy resins into 50% ethanol food simulant during the migration test at 60 °C. Results expressed in µg/dm^2^. Bisphenol A (BPA), Bisphenol A diglycidyl ether (BADGE), Propanol (PrOH). Unknowns 1 and 2 are listed in Paseiro-Cerrato et al. [34].

Days	Unknown 1	Unknown 2	Cyclo-di-BADGE	BADGE + BPA + PrOH	BADGE + 2BPA	BPA	BADGE
0.17	121	72	85	13	52	3	2
0.33	175	114	136	19	87	7	2
1	218	157	236	29	160	7	7
2	246	181	265	32	177	10	8
5	247	188	301	40	185	9	10
10	292	219	314	45	187	12	15
12	267	210	306	45	197	8	12
14	310 ± 9	210 ± 3	314 ± 4	48 ± 1	196 ± 3	12 ± 3	15 ± 1
21	300 ± 18	232 ± 11	286 ± 25	42 ± 4	201 ± 3	11 ± 1	10 ± 3
30	366	258	318	53	206	13	16

**Table 2:** Concentrations of tracked migrants from epoxy resins in in retorted cans containing Miglyol 812^®^ food simulant during the migration test at 60 °C. Results expressed in µg/dm^2^ assuming that 1 Kg is in contact with 6 dm^2^ of the surface of the polymer.

Days	Unknown 1	Cyclo-di-BADGE	BADGE + 2BPA	BPA
2	102	146	109	10
5	100	136	99	7
10	88 ± 6	131 ± 9	87 ± 11	7 ± 2
14	100	139	100	7

**Table 3:** Concentrations of the tracked migrants from acrylic–phenolic coatings into 50% ethanol food simulant during the migration test at 60 °C. Results expressed in µg/dm^2^. ND: not detected.

Days	Unknown B	Unknown E	BGA
0.17	0	8	ND
0.33	15	17	ND
1	20	21	ND
2	28	25	1
5	32	28	2
10	43	35	5
12	43	36	5
14	49 ± 2	38 ± 1	5 ± 1
21	53 ± 1	40 ± 0.1	11 ± 0.4
30	61	41	14

**Table 4:** Concentrations of tracked migrants from polyester coatings into 50% ethanol food simulant at 60 °C. Results expressed in µg/dm^2^. *1, *2, and *3, are listed as IPA + 2 MBO, IPA + MBO + COH and 2 IPA + 2 MBO in Paseiro-Cerrato et al. [33]. Unknowns 5 and 6 are listed in Paseiro-Cerrato et al. [33]. Unknowns A and B are tracked unidentified migrants in the migration test.

Days	IPA + 2MPD*^1^	IPA + MPD + CHDM *^2^	2IPA + 2MPD *^3^	Unknown 5	Unknown 6	Unknown A	Unknown B
2	34	61	11	11	11	11	11
5	51	85	32	42	40	43	59
10	79	140	57	81	86	75	103
12	82	136	55	88	89	80	108
14	97 ± 7	161 ± 17	64 ± 11	99 ± 11	101 ± 14	64 ± 11	112 ± 13
21	169	261	101	173	160	108	137
30	155	271	121	183	157	101	124

**Table 5:** Parameters of LOD, LOQ, correlation coefficient, and repeatability of the studied compounds. BADGE.2H2O was detected at 225 nm while BADGE was detected at 232 nm.

	BGA	BPA	BADGE.2H_2_O	BADGE	BHET
LOD (ng/mL)	0.06	1.5	30	4	80
LOQ (ng/mL)	0.2	5	100	12	240
r^2^	0.999	0.999	0.999	0.999	0.999
% RSD (n = 4–6)	0.9	1.1	0.2	0.4	0.8

## References

[1] Simal-Gandara J., Damant A.P., Castle L. (2002). The use of LC-MS in studies of migration from food contact materials: A review of present applications and future possibilities. Crit. Rev. Anal. Chem..

[2] Paseiro-Cerrato R., Rodriguez-Bernaldo de Quiros A., Sendon R., Bustos J., Santillana M.I., Cruz J.M., Paseiro-Losada P. (2010). Chromatographic methods for the determination of polyfunctional amines and related compounds used as monomers and additives in food packaging materials: A state-of-the-art review. Compr. Rev. Food Sci. Food Saf..

[3] Driffield M., Bradley E.L., Castle L., Coulier L. (2011). Identification of unknown migrants from food contact materials. Methods Mol. Biol..

[4] Hoppe M., de Voogt P., Franz R. (2016). Identification and quantification of oligomers as potential migrants in plastics food contact materials with a focus in polycondensates—A review. Trends Food Sci. Technol..

[5] Silva A.S., Garcia R.S., Cooper I., Franz R., Losada P.P. (2006). Compilation of analytical methods and guidelines for the determination of selected model migrants from plastic packaging. Trends Food Sci. Technol..

[6] Garcia R.S., Silva A.S., Cooper I., Franz R., Losada P.P. (2006). Revision of analytical strategies to evaluate different migrants from food packaging materials. Trends Food Sci. Technol..

[7] Lago M.A., Rodriguez-Bernaldo de Quiros A., Sendon R., Bustos J., Nieto M.T., Paseiro P. (2015). Photoinitiators: A food safety review. Food Addit. Contam. Part A.

[8] Nerin C., Canellas E., Aznar M., Silcock P. (2009). Analytical methods for the screening of potential volatile migrants from acrylic-base adhesives used in food-contact materials. Food Addit. Contam. Part A.

[9] Dutra C., de Alvarenga F.M.T., Nerin C., Bentayeb K., Rodriguez-Lafuente A., Aznar M., Reyes F.G.R. (2014). Migration of residual nonvolatile and inorganic compounds from recycled post-consumer PET and HDPE. J. Braz. Chem. Soc..

[10] Paseiro-Cerrato R., MacMahon S., Ridge C.D., Noonan G.O., Begley T.H. (2016). Identification of unknown compounds from polyester cans coatings that may potentially migrate into food or food simulants. J. Chromatogr. A.

[11] Till D., Schwope A.D., Ehntholt D.J., Sidman K.R., Whelan R.H., Schwartz P.S., Reid R.C. (1987). Indirect food additive migration from polymeric food packaging materials. Crit. Rev. Toxicol..

[12] Schwope A.D., Till D.E., Ehntholt D.J., Sidman K.R., Whelan R.H., Schwartz P.S., Reid R.C. (1987). Migration of BHT and Irganox 1010 from low-density polyethylene (LDPE) to foods and food-simulating liquids. Food Chem. Toxicol..

[13] Figge K. (1980). Migration of components from plastics-packaging materials into packed goods—Test methods and diffusion models. Prog. Polym. Sci..

[14] Till D.E., Ehntholt D.J., Reid R.C., Schwartz P.S., Schwope A.D., Sidman K.R., Whelan R.H. (1982). Migration of styrene monomer from crystal polystyrene to foods and food simulating liquids. Ind. Eng. Chem. Fundam..

[15] Figge K. (1972). Migration of additives from plastic films into edible oils and fat simulants. Food Cosmet. Toxicol..

[16] Figge K. (1973). Determination of total migration from plastics-packaging materials into edible fats using a carbon-14-labeled fat simulant. Food Cosmet. Toxicol..

[17] Begley T.H., Hollifield H.C. (1990). Migration of dibenzoate plasticizers and polyethylene terephthalate cyclic oligomers from microwave susceptor packaging into food-simulating liquids and food. J. Food Prot..

[18] Petersen H.J., Lillemark L., Lund L. (1997). Migration from PVC cling films compared with their field of application. Food Addit. Contam..

[19] Choudhry M.S., Lox F., Buekens A., Decroly P. (1998). Evaluation of migrational behavior of plastic food-contact materials: A comparison of methods. Packag. Technol. Sci..

[20] Komolprasert V., Lawson A.R. (1997). Considerations for Reuse of Poly(ethylene terephthalate) Bottles in Food Packaging: Migration Study. J. Agric. Food Chem..

[21] Aurela B., Ohra-aho T., Soderhjelm L. (2001). Migration of alkylbenzenes from packaging into food and Tenax. Packag. Technol. Sci..

[22] Garde J.A., Catala R., Gavara R., Hernandez R.J. (2001). Characterizing the migration of antioxidants from polypropylene into fatty food simulants. Food Addit. Contam..

[23] Lopez-Cervantes J., Sanchez-Machado D.I., Pastorelli S., Rijk R., Paseiro-Losada P. (2003). Evaluating the migration of ingredients from active packaging and development of dedicated methods: A study of two iron-based oxygen absorbers. Food Addit. Contam..

[24] Tovar L., Salafranca J., Sanchez C., Nerin C. (2005). Migration studies to assess the safety in use of a new antioxidant active packaging. J. Agric. Food Chem..

[25] Kubwabo C., Kosarac I., Stewart B., Gauthier B.R., Lalonde K., Lalonde P.J. (2009). Migration of bisphenol A from plastic baby bottles, baby bottle liners and reusable polycarbonate drinking bottles. Food Addit. Contam. Part A.

[26] Paseiro-Cerrato R., Tongchat C., Franz R. (2016). Study of the partition coefficients Kp/f of seven model migrants from LDPE polymer in contact with food simulants. Food Addit. Contam. Part A.

[27] Bott J., Stoermer A., Franz R. (2014). Migration of nanoparticles from plastic packaging materials containing carbon black into foodstuffs. Food Addit. Contam. Part A.

[28] Sanches Silva A., Cruz Freire J.M., Franz R., Paseiro Losada P. (2008). Time-temperature study of the kinetics of migration of diphenylbutadiene from polyethylene films into aqueous foodstuffs. Food Res. Int..

[29] Garcia Ibarra V., Sendon R., Garcia-Fonte X.-X., Paseiro Losada P., Rodriguez Bernaldo de Quiros A. (2019). Migration studies of butylated hydroxytoluene, tributyl acetylcitrate and dibutyl phthalate into food simulants. J. Sci. Food Agric..

[30] Begley T.H., Hollifield H.C. (1989). Liquid chromatographic determination of residual reactants and reaction by-products in polyethylene terephthalate. J. Assoc. Off. Anal. Chem..

[31] Labuza T.P. (1980). The effect of water activity on reaction kinetics of food deterioration. Food Technol. (Chicago).

[32] Vaclavikova M., Paseiro-Cerrato R., Vaclavik L., Noonan G.O., DeVries J., Begley T.H. (2016). Target and non-target analysis of migrants from PVC-coated cans using UHPLC-Q-Orbitrap MS: Evaluation of long-term migration testing. Food Addit. Contam. Part A.

[33] Paseiro-Cerrato R., Noonan G.O., Begley T.H. (2016). Evaluation of Long-Term Migration Testing from Can Coatings into Food Simulants: Polyester Coatings. J. Agric. Food Chem..

[34] Paseiro-Cerrato R., DeVries J., Begley T.H. (2017). Evaluation of Short-Term and Long-Term Migration Testing from Can Coatings into Food Simulants: Epoxy and Acrylic–phenolic Coatings. J. Agric. Food Chem..

[35] European Commission Commission Regulation(EU) no 10/2011 of 14 January 2011 on Plastic Materials and Articles Intended to Come into Contact with Food. https://www.fsai.ie/uploadedFiles/Reg10_2011.pdf.

[36] Begley T.H., Dennison J.L., Hollifield H.C. (1990). Migration into food of polyethylene terephthalate (PET) cyclic oligomers from PET microwave susceptor packaging. Food Addit. Contam..

[37] Biedermann M., Grob K. (1998). Food contamination from epoxy resins and organosols used as can coatings: Analysis by gradient NPLC. Food Addit. Contam..

[38] Brenz F., Linke S., Simat T. (2018). Linear and cyclic oligomers in polybutylene terephthalate for food contact materials. Food Addit. Contam. Part A.

[39] Eckardt M., Kubicova M., Simat T.J. (2018). Universal response quantification approach using a Corona Charged Aerosol Detector (CAD)–Application on linear and cyclic oligomers extractable from polycondensate plastics polyesters, polyamides and polyarylsulfones. J. Chromatogr. A.

